# 2320: HPA axis predictors of cue-induced intravenous alcohol self-administration in non-dependent drinkers

**DOI:** 10.1017/cts.2017.129

**Published:** 2018-05-10

**Authors:** Honoreé White Brewton, Bethany L. Stangl, Laura E. Kwako, Rajita Sinha, Vijay Ramchandani

**Affiliations:** National Institutes of Health, New York, NY, USA

## Abstract

OBJECTIVES/SPECIFIC AIMS: Alcohol craving, particularly in response to stress and alcohol cues, can lead to relapse in alcohol-dependent individuals. Hypothalamus-pituitary-adrenal (HPA) axis markers such as the cortisol to corticotrophin (CORT:ACTH) ratio have been shown to be a significant predictor of alcohol relapse. Our objective was to evaluate the influence of HPA-axis measures on intravenous alcohol self-administration (IV-ASA) in binge and nonbinge drinkers. METHODS/STUDY POPULATION: Healthy, non-dependent binge drinkers (n=14) and nonbinge drinkers (n=11) participated in this study. They underwent 3 personalized imagery sessions, where they heard 5-minute personalized audio scripts designed to trigger stress, alcohol craving, and neutral-relaxation states. Immediately following these cues, participants were given access to alcohol using a novel IV-ASA paradigm for 120 minutes. Serial blood samples were collected for cortisol and ACTH levels. Subjective measures were collected serially using the Subjective Units of Distress Scale (SUDS), Drug Effects Questionnaire (DEQ), and Alcohol Urge Questionnaire (AUQ). Analyses were conducted using linear regression. RESULTS/ANTICIPATED RESULTS: Results showed that peak and average ACTH levels as well as the CORT:ACTH ratio during the early phase of the IV-ASA session following the stress and alcohol cues were significantly higher than the neutral script; this effect was seen primarily in binge drinkers. After script administration, a greater change from baseline for ACTH predicted time to peak BrAC during IV-ASA. Gender and binge group predicted AUQ MAX (peak alcohol craving over the entire study session) and WANT MAX (peak “want more alcohol” scores over the session). There was a significant correlation between IV-ASA and increased ACTH peak and average values in binge drinkers. The DEQ and AUQ measures were positively correlated with ACTH peak and ACTH change from baseline. DISCUSSION/SIGNIFICANCE OF IMPACT: These findings, to our knowledge, are the first demonstration that exposure to both stress and alcohol cues lead to an increase in ACTH during cue-induced IV-ASA, particularly in binge drinkers. These results suggest that changes in HPA-axis reactivity following stress and alcohol may be important determinants of alcohol consumption in non-dependent binge drinkers.

